# NF-κB Signaling in Targeting Tumor Cells by Oncolytic Viruses—Therapeutic Perspectives

**DOI:** 10.3390/cancers10110426

**Published:** 2018-11-08

**Authors:** Justyna Struzik, Lidia Szulc-Dąbrowska

**Affiliations:** Division of Immunology, Department of Preclinical Sciences, Faculty of Veterinary Medicine, Warsaw University of Life Sciences-SGGW, Ciszewskiego 8, 02-786 Warsaw, Poland; lidia_szulc@sggw.pl

**Keywords:** immunotherapy, NF-κB signaling, oncolytic viruses

## Abstract

In recent years, oncolytic virotherapy became a promising therapeutic approach, leading to the introduction of a novel generation of anticancer drugs. However, despite evoking an antitumor response, introducing an oncolytic virus (OV) to the patient is still inefficient to overcome both tumor protective mechanisms and the limitation of viral replication by the host. In cancer treatment, nuclear factor (NF)-κB has been extensively studied among important therapeutic targets. The pleiotropic nature of NF-κB transcription factor includes its involvement in immunity and tumorigenesis. Therefore, in many types of cancer, aberrant activation of NF-κB can be observed. At the same time, the activity of NF-κB can be modified by OVs, which trigger an immune response and modulate NF-κB signaling. Due to the limitation of a monotherapy exploiting OVs only, the antitumor effect can be enhanced by combining OV with NF-κB-modulating drugs. This review describes the influence of OVs on NF-κB activation in tumor cells showing NF-κB signaling as an important aspect, which should be taken into consideration when targeting tumor cells by OVs.

## 1. Introduction

Since its discovery in 1986, nuclear factor (NF)-κB has been widely studied and is well known as a pleiotropic transcription factor, which orchestrates inflammation, innate and adaptive immune responses, cell growth, and apoptosis. Therefore, deregulation of NF-κB activation pathways may result in autoimmune diseases and tumorigenesis. Importantly, NF-κB and its target genes have been shown to play a role in malignant transformation, proliferation, and survival of cancer cells, as well as angiogenesis and invasion/metastasis. Moreover, aberrant NF-κB signaling is associated with aggressiveness, recurrence, and therapeutic resistance of tumors. Therefore, NF-κB is considered a good candidate for the therapeutic target, becoming an important focus in cancer research [1,2,3,4].

Chemotherapy, which is widely used for cancer treatment, is mainly aimed at apoptosis, necrosis, autophagy, and mitotic catastrophe. Importantly, cellular senescence, resulting from chemotherapy treatment, involves NF-κB activation. This suggests that blockage of NF-κB would prevent this outcome. Unfortunately, due to the pleiotropic nature of NF-κB, the use of NF-κB inhibitors in cancer therapy may not always be beneficial. Apoptotic defects of cancer cells are responsible for chemoresistance. Therefore, these factors should be considered when targeting NF-κB in cancer treatment [1].

## 2. Overview of NF-κB Signaling

NF-κB is a family of transcription factors, which is composed of the following proteins: RelA/p65, RelB, c-Rel, NF-κB1/p105, and NF-κB2/p100. In resting cells, NF-κB localizes mainly to the cell cytoplasm as RelA/p50 heterodimers coupled with an inhibitor of κB (IκB). Other heterodimers comprise RelB and p100, a p52 precursor protein, which acts in a similar manner as IκB, maintaining the state of the cytoplasmic arrest of NF-κB [4,5]. p105 and p100 phosphorylation result in their 26S proteasome-induced processing via the activity of Skp-Cullin-F-box (SCF) ubiquitin E3 ligase. These events generate p50 and p52 active subunits allowing nuclear translocation of heterodimers comprising RelA/p50 or RelB/p52 and the transcription of target genes [5].

**Figure 1:**
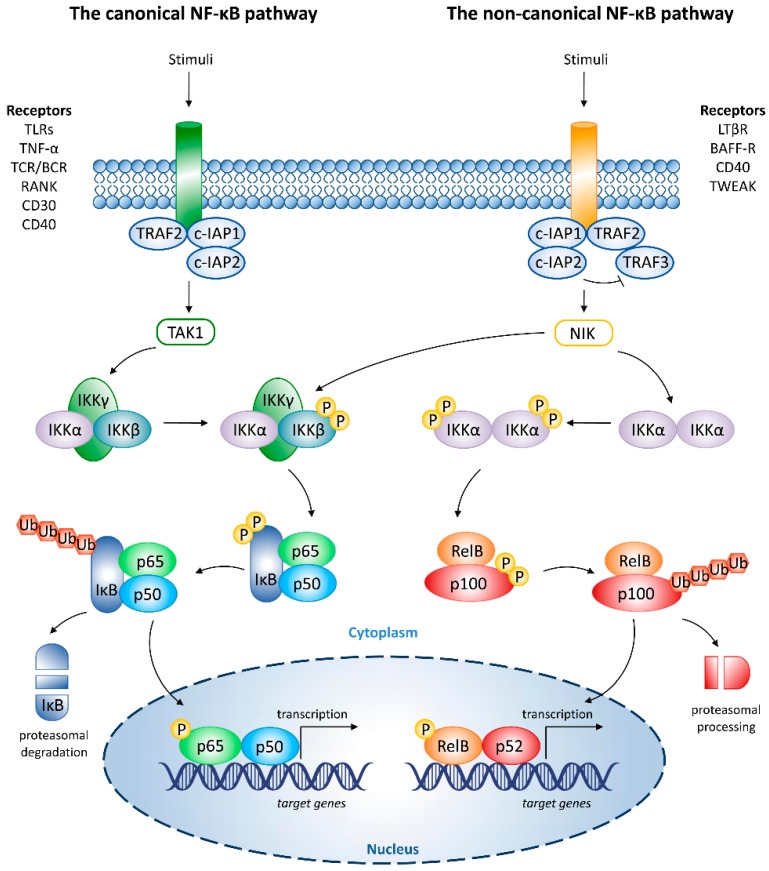
Canonical and non-canonical nuclear factor (NF)-κB signaling. Activation and inhibition of NF-κB signaling components are indicated by pointing and blunt arrows, respectively. BAFF-R, B cell-activating factor receptor; BCR, B-cell receptor; CD30, cluster of differentiation 30; CD40, cluster of differentiation 40; c-IAP1, cellular inhibitor of apoptosis protein-1; c-IAP2, cellular inhibitor of apoptosis protein-2; IKKα, inhibitor of κB kinase α; IKKβ, inhibitor of κB kinase β; IKKγ, inhibitor of κB kinase γ; IκB, inhibitor of κB; LTβR, lymphotoxin-β receptor; NIK, nuclear factor-κB-inducing kinase; P, phosphate group; RANK, receptor activator of nuclear factor-κB; TAK1, transforming growth factor-β-activated kinase 1; TCR, T-cell receptor; TLRs, Toll-like receptors; TNF-α, tumor necrosis factor-α; TRAF2, tumor necrosis factor receptor-associated factor 2; TRAF3, tumor necrosis factor receptor-associated factor 3; TWEAK, tumor necrosis factor-like weak inducer of apoptosis; Ub-ubiquitin moieties.

Canonical (classical) NF-κB signaling pathway, which results in nuclear translocation of RelA/p50 and c-Rel/p50 dimers, is triggered by certain receptors activated by proinflammatory cytokines, such as tumor necrosis factor (TNF)-α. Other stimuli of canonical NF-κB signaling, such as lipopolysaccharide (LPS), belong to pathogen-associated molecular patterns (PAMPs), which activate Toll-like receptors (TLRs). Canonical NF-κB activation pathway is also triggered by activators of receptors of B- and T cells (BCR and TCR), stimulatory molecules of receptor activator of NF-κB (RANK), as well as a cluster of differentiation 30 (CD30) and CD40 ligands (Figure 1) [4]. Cellular receptors bind cellular inhibitor of apoptosis protein-1 (c-IAP1) and c-IAP2, which cooperate with TNF receptor-associated factor 2 (TRAF2) adapter protein. Following canonical NF-κB activation, IκB kinase complex (IKK), comprising IKKα, IKKβ, and IKKγ/NF-κB essential modulator (NEMO) components, is activated by transforming growth factor-β-activated kinase 1 (TAK1). IKK activation triggers phosphorylation, ubiquitination, and proteasomal degradation of IκB. These events enable the release and nuclear translocation of predominantly p65/p50 heterodimers. Canonical NF-κB activation, which does not depend on protein synthesis, is described as rapid and transient. Canonical NF-κB signaling controls cell survival, innate immunity, regulation of the type I interferon (IFN) antiviral response, and inflammation [6,7].

Non-canonical (alternative) NF-κB activation pathway is typically induced by TNF receptor (TNFR) superfamily, including lymphotoxin-β receptor (LTβR), B cell-activating factor receptor (BAFF-R), CD40, and TNF-like weak inducer of apoptosis (TWEAK) (Figure 1) [7,8]. In unstimulated cells, NF-κB-inducing kinase (NIK) undergoes persistent degradation by TRAF3. Upon receptor stimulation, c-IAP, c-IAP2, and TRAF2 degrade TRAF3. As a result, NIK is stabilized, inducing IKKα phosphorylation. Afterward, RelB-associated NF-κB2 p100 undergoes NIK/IKKα-induced phosphorylation and proteasomal processing [6,7,8,9,10]. Finally, in order to control the transcription of genes responsible for adaptive immunity, encompassing immune organ and B-cell development, dendritic cell (DC) activation, and bone metabolism, RelB/p52 complexes translocate to the nucleus. Non-canonical NF-κB signaling is slow, persistent, and relies on protein synthesis [7,8]. Alterations in the non-canonical NF-κB activation are linked to inflammation, autoimmune diseases, lymphoid malignancies, and osteoporosis [7]. Despite different functions, canonical and non-canonical NF-κB signaling are linked due to cross-talk mechanisms [11]. The link between the two signaling pathways may also exist via the activity of NIK kinase, which may stimulate both non-canonical and canonical NF-κB signaling pathways [12].

## 3. NF-κB in Oncogenesis

NF-κB’s role in oncogenesis was first identified when v-rel oncogene, a c-Rel homolog, expressed by reticuloendotheliosis virus strain T, was identified as the key etiological agent of fatal lymphomas in chicken models [4]. Constitutive NF-κB activation is linked to cancer development and can be observed in lymphoid malignancies and tumors of epithelial origin [4,13]. NF-κB’s role in cancer is multidirectional because genes encoding both pro-proliferative and anti-apoptotic proteins are under NF-κB’s control. Furthermore, NF-κB signaling is linked to other pathways, including activator protein 1 (AP1), signal transducer and activator of transcription 3 (STAT3), IFN-regulatory factor, Notch, NF-E2-related factor 2 (NRF2), p53, and WNT–β-catenin signaling [14].

If regulatory T (Treg) cells or myeloid-derived suppressor cells (MDSCs) are present in the tumor microenvironment, NF-κB acts as an inhibitor of antitumor responses. Adversely, the role of NF-κB in antitumor immunity leans on inflammatory cells, including perforin-secreting natural killer (NK) cells and tumor-phagocytic macrophages [4]. Nonetheless, chronic inflammation and fibrosis linked to NF-κB stimulation create a tumorigenic microenvironment [13]. In order to upregulate expression of genes encoding cyclooxygenase (COX)-2, nitric oxidase synthase, and proinflammatory cytokines, such as TNF-α, interleukin (IL)-6, -8, and chemokine (C-C motif) ligand (CCL)-2 in immune cells, IL-1, TNF, PAMPs, and damage-associated molecular patterns (DAMPs) activate NF-κB. These molecules, in turn, stimulate NF-κB and STAT3 in tumor cells and surrounding tissues to promote cancer survival, invasion and metastasis [14]. Importantly, RelA-dependent chemokine (C-X3-C motif) ligand 1 (CX3CL1), which recruits cytotoxic T lymphocytes (CTLs) and NK cells and exerts an antitumor effect, supports TNF-related apoptosis-inducing ligand (TRAIL) resistance and the survival of pancreatic ductal adenocarcinoma (PDAC) cells [15]. Similarly, CCL20, produced by PDAC cells, attracts peripheral blood mononuclear cells (PBMCs) to increase TRAIL resistance of pancreatic cancer cells [16].

NF-κB controls epithelial-mesenchymal transition and the expression of cell adhesion molecules (CAMs), such as E-selectin, intracellular CAM (ICAM)-1, vascular CAM (VCAM)-1, and integrins [14,17]. However, inhibition of NF-κB may not be beneficial for anticancer therapy since angiogenesis is antagonized by NF-κB. In addition, tumor invasion of adjacent tissues is associated with NF-κB-dependent matrix metalloproteinases (MMPs) [3,14]. NF-κB is also involved in metabolism switch from oxidative phosphorylation to glycolysis [14].

Suppression of intrinsic and extrinsic apoptotic pathways by NF-κB may arise from upregulation of IAP family members upon TNFR stimulation. IAPs not only inhibit caspase activation, but may also stimulate NF-κB, thus enhancing the anti-apoptotic effect. Indeed, in cancer cells, IAPs’ overexpression can be observed [13]. Cell survival and resistance to anticancer therapeutics can also be achieved via growth factors and their receptors, such as epidermal growth factor receptor, and other factors, for example, rat sarcoma (Ras) oncoprotein. TRAF family member-associated NF-κB activator-binding kinase 1 (TBK1), activated downstream of Ras and growth factor receptors, stimulate NF-κB via c-Rel. NF-κB can also be activated by breakpoint cluster region (Bcr)-Abelson leukemia oncogene (Abl) fusion protein, associated with chronic myeloid leukemia and acute lymphoblastic leukemia, via activation of IKKs. Importantly, in promoting both NF-κB-dependent and -independent cell survival, IKKs are regarded as key players [2].

Elevated IKK activation can be observed in leukemia, lymphoma, multiple myeloma (MM), as well as in breast cancer, colorectal cancer, and prostate cancer. IKK expression is also correlated with cell survival in clear cell renal cell carcinoma (CCRCC) [18,19]. Importantly, silencing IKK subunits using small interfering RNA (siRNA) or treatment with IKK inhibitor helps sensitize cancer cells to chemotherapy and trigger cell death [18].

The role of NF-κB in replicative immortality involves upregulation of the telomerase catalytic subunit (TERT)-encoding gene, whereas the expression of NF-κB-dependent genes is regulated by TERT [17]. Also, genetic instability can be acquired via the DNA damage induced by oxidative stress, which activates NF-κB [2,17]. NF-κB’s direct role in cancer is based on the mutations of NF-κB regulatory proteins. Mostly, gain-of-function mutations of upstream NF-κB activators are responsible for NF-κB-driven cancers [2,4,17]. In addition, genetic and chromosomal alterations of *NF-KB1*, *NF-KB2*, and *REL* are typical for a variety of types of blood cancers [2,17].

Oncogenic transformation is also linked to certain viral factors, which act antiapoptotically via persistent activation of NF-κB signaling in the host cells. The canonical and non-canonical NF-κB signaling pathways are induced via TRAFs by Epstein-Barr virus (EBV)-encoded latent membrane protein 1 (LMP1), leading to Hodgkin’s lymphoma. IKKs can be stimulated by Tax oncogene of human T-cell leukemia virus type 1 (HTLV-1), a causative agent of adult T-cell leukemia. Kaposi’s sarcoma-associated herpesvirus (KSHV) activates IKKγ via anti-apoptotic protein viral FLICE inhibitory protein (vFLIP) [2,4,7,20].

Since apoptotic stimuli, such as proinflammatory TNF, chemotherapeutic daunorubicin, as well as ionizing radiation may be responsible for the anti-apoptotic role of NF-κB, it is important to inhibit NF-κB during cancer treatment to overcome tumor resistance. This approach of selective NF-κB inhibition can be used in gastric cancer chemotherapy, as well as in melanoma doxorubicin treatment, which is performed together with IKK inhibition [17]. Upon targeted NF-κB inhibition, TRAIL-induced cancer cytotoxicity is observed [17,21]. It is also worth noticing that TNF superfamily members, for example, TWEAK, activate NF-κB-dependent TNF expression resulting in cell death. Thus, NF-κB may act proapoptotically [21].

## 4. OVs

OVs, belonging to new generation of cancer immunotherapeutics, are natural or genetically modified pathogens, which infect and replicate in cancer cells but not in non-transformed cells, and trigger both antiviral and antitumor responses [22]. Upon administration of OV, the virus infects tumor cells resulting in their lysis. As a consequence of tumor-derived antigens’ (TDAs) release, antigen-presenting cells (APCs) uptake and process TDAs to activate and prime T cells. Thus, the effector cells localize to, infiltrate, and eventually kill the tumor cells. Afterward, released TDAs are processed by APCs [23]. Nevertheless, using OVs as monotherapy may not be efficient due to the limited replication of the virus in the host, tumor resistance to the response generated, and immunosuppression within the tumor microenvironment [22].

In oncolytic virotherapy, one of the main concerns is the presence of neutralizing antibodies, which can already be found in patients vaccinated or previously treated with OVs [24,25]. This effect can be observed in MM patients treated with systemically administered measles virus armed with human thyroidal sodium iodide symporter (MV-NIS) [24]. Upon intravenous delivery of OV, both antibodies and complement promote Fc receptor-linked clearance of the virus by Kupfer cells and splenic macrophages [25]. However, such administration is not always beneficial. For instance, oncolytic herpes simplex virus type 1 (HSV)-1, which spreads from cell to cell, and is used for melanoma treatment, is more effective when administered intralesionally [24]. Nevertheless, intratumoral injection of an OV may not be efficient in the treatment of disseminated tumors, whereas systemic administration of a drug in trans with OV delivery may result in toxicity and increases the costs. Adversely, delivery of therapeutic gene product or a single therapeutic in cis may not be efficient when sustained expression is required [26]. Therefore, many therapeutic approaches based on OVs are under clinical trials. Nevertheless, the United States Food and Drug Administration (FDA) approved Talimogene laherparepvec (T-VEC), a modified HSV, in metastatic melanoma treatment [22,23,27,28]. In clinical trials, metastatic melanoma patients are given intralesional injections of T-VEC combined with intravenous pembrolizumab (anti-programmed death [PD]-1). This treatment represents a strategy of switching immunologically “cold” tumor, which is characterized by the absence or low tumor-infiltrating lymphoid cells (TILs), into “hot.” The latter is defined by the presence of TILs in their microenvironment due to induction of proinflammatory response and activation of DCs and NK cells [22,29].

TILs are represented by subpopulations of CD3^+^ CD4^+^ helper and CD3^+^ CD8^+^ cytotoxic T cells [30], Tregs, NK cells, B cells, DCs, macrophages, and MDSCs [31]. TILs comprise epithelial, stromal, and peritumoral lymphocytes located in cancer cell nests, central stroma, and invasive margins, respectively. Although TILs may display higher reactivity toward tumor cells compared with non-infiltrating lymphocytes, they may promote tumor growth. Importantly, the presence and immunophenotype of TILs serve as a prognostic factor in melanoma, breast cancer, ovarian cancer, lung cancer, and renal cell carcinoma (RCC). For instance, in advanced melanoma, TILs Treg CD4^+^CD25^+^ are linked to progression of the disease [30].

TILs obtained from resected tumors are used as effector cells in adoptive T-cell therapy (ATC), which can be combined with chemotherapy and immunotherapeutic drugs to maintain TILs proliferation and proper function to target tumor-associated antigens (TAAs). After ex vivo propagation and activation, TILs and IL-2, a T-cell growth factor, are administered autologously to lymphodepleted patients [30,32,33]. To facilitate virus entry to the tumor, TILs may be genetically modified with C-X-C chemokine receptor type 2 (CXCR2) [34]. The improvement of antitumor activity in vitro can also be obtained by blockade of transforming growth factor (TGF)-β signaling [35]. With durable responses being observed, TILs have been implemented in late-stage metastatic melanoma in clinical studies [33]. Clinical studies are currently carried on TILs combined with anti- CTL-associated protein (CTLA)-4 monoclonal antibodies (mAbs) (ipilimumab) (NCT01701674), peginterferon α-2b upregulating human leukocyte antigen (HLA) expression on tumor cells (NCT02379195), and nivolumab (anti-PD-1 mAbs) with or without IFN-α (NCT03638375) for metastatic melanoma treatment. Other clinical trials implementing TILs are focused on the treatment of nasopharyngeal carcinoma (NCT02421640), malignant pleural mesothelioma (NCT02414945), cervical carcinoma (NCT03108495), and squamous cell carcinoma of the head and neck (NCT03083873). Also, TILs along with pembrolizumab and other drugs are tested against digestive tract, urothelial, breast, ovarian/endometrial tumors (NCT01174121). For non-small cell lung cancer (NSCLC), TILs with durvalumab (anti-PD-ligand 1 (PD-L1) mAbs) (NCT03419559), nivolumab, and other drugs are under clinical trials (NCT03215810). TILs are also tested in treatment of uveal neoplasms (NCT03467516), platinum-resistant high-grade serous ovarian, fallopian tube, or primary peritoneal cancer (NCT01883297).

The expression of PD-1 on T cells is important in ATC. PD-1 binds tumor-associated PD-L1 and PD-L2, which interfere with T-cell activation, major histocompatibility complex (MHC), and TCR interaction, and induces T-cell apoptosis. Other immune checkpoint protein, CTLA-4 suppresses T-cell activation. Therefore, to induce the immune response, CTLA-4 or PD-1 interactions with ligands can be disrupted by mAbs in cancer immunotherapy [30].

In metastatic melanoma, injection of T-VEC results in local inflammation, the presence of type I IFNs, and granulocyte-macrophage-colony-stimulating factor (GM-CSF)-attracted DCs, leading to subsequent cell killing. Anti-PD-1 blocks the interaction between PD-1 on activated T cells and PDL-1 on tumor cells. Thus, T cells are reactivated to destroy the tumor [29]. Another target in antitumor therapy, CTLA-4, which can be expressed on tumor cells, infiltrating Tregs as well as on exhausted conventional T cells, is regarded as an immunosuppressive factor, but its role as a prognostic factor is unclear [36]. In advanced melanoma stage IIIc and IV treated with intratumoral injections of coxsackievirus A21 (CVA21), which infects ICAM-1 expressing cancer cells, antitumor response has been shown in clinical trials. Importantly, in preclinical studies on a mouse model, the use of anti-PD-1 or anti-CTLA-4 mAbs together with CVA improved anti-tumor response [37].

Certain viral vectors described below and derived from oncolytic DNA (adenovirus (AV), HSV-1, parvovirus (PV)) and RNA viruses (reovirus (RV) and vesicular stomatitis virus (VSV)) presented in this review are currently under clinical trials (Table 1).

**Figure 2:**
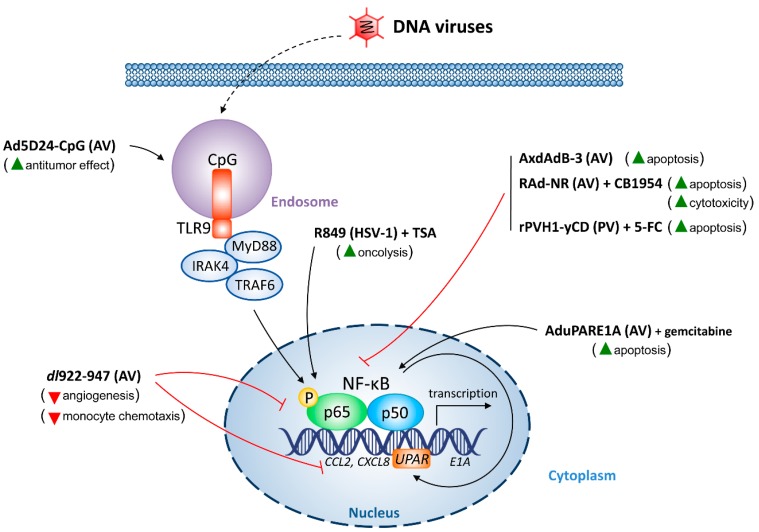
The impact of oncolytic DNA viruses on nuclear factor (NF)-κB signaling. Activation of NF-κB signaling components is indicated by pointing black arrows. Inhibition of NF-κB is indicated by red blunt arrows. The outcomes of NF-κB modulation are shown in the brackets. Induction is indicated by green triangles, whereas inhibition—by inverted red triangles. 5-FC, 5-fluorocytosine; AV, adenovirus; *CCL2*, chemokine (C-C motif) ligand 2 gene; CpG, cytosine:guanine islands; *CXCL8*, C-X-C motif chemokine ligand 8 gene; HSV-1, herpes simplex virus type 1; IRAK4, interleukin-1 receptor–associated kinase 4; MyD88, myeloid differentiation primary response protein 88; PV, parvovirus; TLR9, Toll-like receptor 9; TRAF6, tumor necrosis factor receptor-associated factor 6; TSA, trichostatin A; *UPAR*, urokinase-type plasminogen activator receptor promoter.

**Figure 3:**
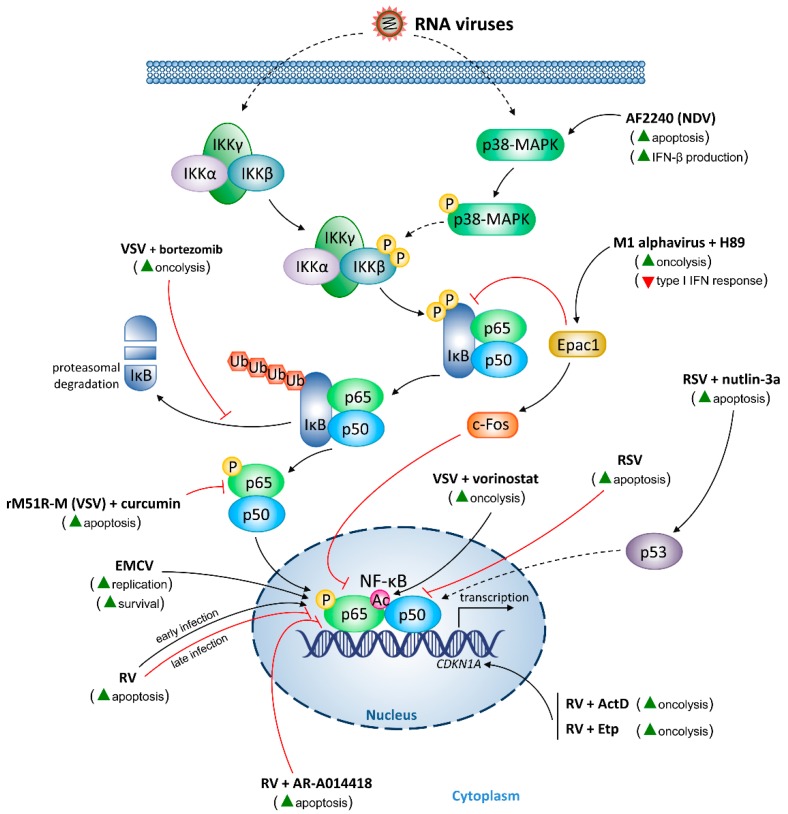
Modulation of nuclear factor (NF)-κB signaling by oncolytic RNA viruses. Pointing black arrows indicate activation of NF-κB signaling components, whereas red blunt arrows indicate NF-κB inhibition. The effects of therapeutic agents on NF-κB are shown in the brackets. Green triangles represent induction, whereas inverted red triangles indicate inhibition. Ac, acetyl group; ActD, actinomycin-D; *CDKN1A*, cyclin-dependent kinase inhibitor 1 gene; c-Fos, cellular oncogene Fos; EMCV, encephalomyocarditis virus; Epac1, exchange protein directly activated by cyclic adenosine monophosphate isoform 1; Etp, etoposide; IFN-β, interferon-β; IKKα, inhibitor of κB kinase α; IKKβ, inhibitor of κB kinase β; IKKγ, inhibitor of κB kinase γ; IκB, inhibitor of κB; NDV, Newcastle disease virus; P, phosphate group; p38-MAPK, p38 mitogen-activated protein kinase; RSV, respiratory syncytial virus; RV, reovirus; Ub, ubiquitin moieties; VSV, vesicular stomatitis virus.

In tumor cells, the cellular response toward oncolytic DNA (Figure 2) and RNA viruses (Figure 3) is influenced by NF-κB. Since NF-κB is a key player in tumorigenesis, NF-κB activation level may indicate the effectiveness of oncolytic virotherapy.

## 5. OVs and NF-κB

### 5.1. DNA Viruses

#### 5.1.1. AV

AVs, belonging to *Adenoviridae* family, are species-specific double-stranded (ds) DNA viruses, which infect humans and other vertebrates [38]. When used as OVs, AVs may induce unwanted dominant antiviral immune response [25,38,39]. Importantly, the stroma of target tumor blocks the diffusion of the virus, and neutralizing antibody production can be observed even after the intratumoral injection of the virus. Therefore, AV-based oncolytic virotherapies need improvement [25,38].

Nevertheless, the China State Food and Drug Administration approved oncolytic AV, H101 (Oncorine) (Shanghai Sunway Biotech), lacking E1B-55K and E3B proteins, for head and neck cancer therapy [40]. E1B-55K inhibits p53-induced apoptosis, thus playing a protective role against E1A interactions with p53 [41], and influences transport and stabilization of mRNA in the cytoplasm [42]. In addition, genes encoding 6.7K, gp19K, 11.6K E3 proteins, which trigger apoptosis, and therefore may limit viral replication, have been deleted [40].

Another AV, DNX-2401 (DNAtrix), with partial *E1A* gene deletion in retinoblastoma (Rb)-binding domain and integrin receptor arginine-glycine-aspartic acid (RGD)-4C insertion, which may act against glioma, was designed by FDA as an orphan drug. ONCOS-102 (Oncos therapeutics) with Δ24 deletion within Rb-binding *E1A* gene, insertion of GM-CSF-encoding gene and replacement of serotype 3 AV knob protein was also granted by FDA as an orphan drug against ovarian cancer, glioma, and malignant mesothelioma [42,43,44]. ONCOS-102 was tested in preclinical peritoneal mesothelioma model [45], and its efficiency in phase I clinical trials in the treatment of ovarian cancer has been demonstrated [46]. ONCOS-102 expressing GM-CSF is currently under phase I of clinical trials on malignant solid tumors treatment and is likely to prolong the survival of patients with ovarian carcinoma and malignant pleural mesothelioma [47].

The importance of E1A Rb-binding domain in NF-κB regulation has been demonstrated on AV vector AxdAdB-3. AxdAdB-3 is characterized by mutated Rb-binding domain of E1A and lack of E1B-55K and is derived from E1B-55K-deficient AxE1AdB [48]. AxdAdB-3 profoundly reduced NF-κB activity and, therefore, increased NF-κB-mediated apoptosis in comparison with AxE1AdB parent vector in human esophageal carcinoma EC-GI-10 cell line [49].

Oncolytic AV type 5, dl922-947 mutant with deletions in E1A-conserved region 2 (CR2) and subsequent Rb-binding deficiency [50], is a candidate for anaplastic thyroid carcinoma treatment. In 8505-c and BHT101-5 human thyroid carcinoma anaplastic cell lines and TPC1 papillary cells, dl922-947 decreased C-X-C motif chemokine ligand 8 (*CXCL8*) promoter binding by p65. In addition, dl922-947 reduced *CCL2* promoter binding by NF-κB in 8505-c and TPC1 cells by displacing p65 from the promoters. In vitro experiments have shown that the reduction of *CXCL8* and *CCL2* expression is linked to impaired tumor-induced angiogenesis and reduced chemotaxis. In vivo experiment on anaplastic thyroid carcinoma xenograft mouse model revealed that dl922-947 reduces *CXCL8* expression and angiogenesis [51].

Conditionally replicating AV, AduPARE1A, harboring urokinase-type plasminogen activator receptor (*UPAR*) promoter, which controls E1A region, may act against pancreatic cancer [52]. In human primary pancreatic adenocarcinoma BxPC-3 and pancreas ductal adenocarcinoma PANC-1 cell lines, the synergistic antitumor effect of gemcitabine and AduPARE1A on NF-κB was demonstrated. The *UPAR* promoter is activated by NF-κB, which, in turn, is stimulated by gemcitabine. Therefore, NF-κB induces *UPAR*-controlled transgenes, and increase in *E1A* expression is observed. Competition between the adenoviral promoter and cellular promoters of NF-κB-regulated anti-apoptotic genes may lead to cell sensitization to gemcitabine-induced apoptosis [53].

Another important AV factor, E1B 19K, is a counterpart of mammalian B-cell lymphoma (Bcl)-2, which counteracts E1A-induced apoptosis and interferes with Bad and Bax proapoptotic proteins [54]. It has been shown that *E1*-deleted replication-defective AV harboring nitroreductase (NR)-encoding gene (RAd-NR) activates IκBα phosphorylation and NF-κB p65/p50 heterodimers in SKOV3 human ovarian carcinoma cell line. In addition, inhibition of NF-κB resulted in restored chemosensitivity of SKOV3 cells because of increased apoptosis. Also, RAd-NR induced NF-κB-dependent mRNA levels of *c-IAP1* and *c-IAP2*, as well as proinflammatory IL-6 secretion. Moreover, conditionally replicating oncolytic *E1B*-attenuated dl1529 or CR-NR induced NF-κB activation and increased apoptotic threshold in HeLa cells. Importantly, inhibition of NF-κB in SKOV3 cells resulted in enhanced cytotoxic effect and increased apoptosis upon RAd-NR/CB1954 virus-directed enzyme prodrug therapy with the delivery of CB1954 prodrug-activating NR [55].

The E1A 243R and p53-binding E1B 55K AV proteins are known as inhibitors of inflammation upon virus infection, whereas E1B 19K counteracts inflammation in the presence of the two E1A 243R and E1B 55K [56]. Moreover, it has been demonstrated that cytopathic effect (CPE) resulting from wild-type (wt) AV infection is nonapoptotic. However, human lung adenocarcinoma A549 cells that underwent apoptosis upon infection with Ad5 mutant with E1B 19K-encoding gene deletion (H5dl337) were unable to inhibit NF-κB activation and proinflammatory cytokine responses in macrophages [57].

In order to enhance the antitumor effect, NF-κB activation can be induced via TLR9 by oncolytic Ad5D24-CpG, containing cytosine:guanine (CpG) islands within the genome. Importantly, stimulation of TLR9 induces DCs to type I IFN-mediated activation and proinflammatory IL-12 secretion. These events, in turn, promote NK cell cytotoxicity and IFN-γ secretion to induce antitumor responses. Such observations have been made in A549 lung cancer xenografts model. Also, a syngeneic model of melanoma showed an enhanced tumor response, including a profound drop in both total number and activation of MDSCs [58]. The efficacy of Ad5D24-CpG was also tested in mouse model of melanoma over-expressing chicken ovalbumin (OVA) [59]. Ad5D24-CpG delivering antiproliferative L-carnosine reduced lung and colon tumor growth in mouse xenograft model [60]. Also, in mouse xenograft model of human lung cancer, tumor growth was reduced by using Ad5D24-CpG in combination with paclitaxel [61].

#### 5.1.2. HSV

HSV-1, belonging to family *Herpesviridae* of neurotropic dsDNA viruses, is a causative agent of a self-limiting disease. Due to its cytolytic replication cycle and easily modified large genome, HSV-1 is beneficial as an oncolytic agent [62,63]. Moreover, HSV-1 infects different cell types, and the presence of a separate attachment and fusion glycoproteins within its envelope is beneficial for modification to improve tumor targeting [63].

The US FDA and European Medicines Agency approved T-VEC (IMLYGIC, Amgen) of GM-CSF-expressing HSV-1 with deletion of *γ_1_34.5* gene encoding infected cell protein (ICP)34.5 neurovirulence factor and ICP47-encoding *α47* gene deletion in inoperable stage IIIb to IV melanoma. Also, FDA granted G207 with *γ_1_34.5* and large subunit of ribonucleotide reductase (*UL39*) gene deletions (MediGene AG, Martinsried, Germany) as an orphan drug in glioma treatment [44,64].

NF-κB activation might be critical for HSV-1 replication as shown in HT29, SW480 human colon carcinoma and Capan2 human pancreatic cancer cells. Nevertheless, NF-κB-inducing chemotherapy agents, such as TNF-α, 5-fluorouracil (5-FU), and irinotecan (CPT-11), inhibited viral replication in the colon and pancreatic cancer cells infected with HSV-1 KOS strain (HSV-1 KOS). Importantly, 5-FU, CPT-11, but not methotrexate (MTX), activate NF-κB in HT29 cells. MTX, which inhibits HSV-1 replication, may act via NF-κB inhibition. Therefore, chemotherapeutic agents, which activate NF-κB, are not beneficial for HSV-1-induced oncolysis [65].

However, the enhancement of HSV-1 oncolytic activity can be obtained by combining HSV-1 with trichostatin A (TSA) [66]. TSA is an antitumor agent belonging to a histone deacetylase (HDAC) inhibitors, which change chromatin structure and act against skin cancer cells [67] and induce apoptosis [68]. For *γ_1_34.5* gene-deficient HSV-1 mutant R849 with *lacZ* encoding bacterial β-galactosidase substitution [69], it has been demonstrated that TSA, which enhances p65 acetylation and nuclear accumulation, promotes viral replication in oral squamous cell carcinoma (SCC) SAS cells. This effect results from TSA-driven enhancement of DNA binding by NF-κB during HSV-1 infection. As a consequence, the oncolytic activity of HSV-1 toward SCC can be improved [66].

The improvement of oncolytic HSV activity is also needed for malignant peripheral nerve sheath tumors (MPNSTs), which are highly aggressive and may be associated with neurofibromatosis type 1 (NF1). Therefore, preclinical models are being developed [70]. Some therapeutic approaches may utilize NF-κB inhibitors in MPNSTs, where NF-κB is constitutively active and cells are resistant to oncolytic HSV. The possible role of NF-κB signaling in IFN-stimulated genes (ISGs) expression has led to the conclusion that using NF-κB inhibitors, such as TPCA-1, which decrease ISGs expression, may improve productive infection of oncolytic HSV Δ*γ_1_34.5* recombinants, which are devoid of ICP34.5 neurovirulence factor and whose replication is limited by protein kinase R (PKR) [71].

Another antitumor approach is based on UV-inactivated HSV-1 (UV-HSV-1), which is proposed for use as an adjuvant in donor mononuclear or NK cell infusions in acute myeloid leukemia therapy. UV-HSV-1 may induce cytolytic activity of human PBMCs and NK cells via TLR2/protein kinase C (PKC)/NF-κB, resulting in p65 nuclear translocation. This treatment results in leukemic cell killing [72].

#### 5.1.3. PV

H-1 protoparvovirus (H-1PV), a member of *Parvoviridae* family, is a single-stranded (ss) DNA rodent pathogen and a promising OV, which can be administered by different routes. The main advantage of H-1PV is that it can cross the blood-brain barrier. H-1PV, whose replication is S-phase-dependent, evokes proinflammatory responses and is a hope for therapies of central nervous system tumors, including glioblastoma [73]. H-1PV armed with IL-2 can be used against lymphoma, glioma, colon, gastric cancer, neuroectodermal, pancreatic cancer, and others [25,73].

H-1PV’s role in oncolytic therapy could rely on immune priming and influencing maturation of DCs to exert antitumor immunity [74]. Human interdigitating DCs (iDCs), incubated with H-1PV-induced tumor cell lysates, exhibited increased expression of TLR3, TLR9, and NF-κB and produced higher amounts of TNF-α compared to uninfected human melanoma (SK29Mel) cells in ex vivo model [75].

In oncolytic therapy of pancreatic cancer, strategies of gene transfer involve introduction of a suicide therapeutic gene, which is missing or underexpressed in tumor cells. This approach allows obtaining a bystander effect of antitumor response toward neighboring cells [76]. Recombinant parvovirus rPVH1-yCD expressing the suicide gene *yCD* (yeast cytosine deaminase), which converts the prodrug 5-fluorocytosine (5-FC) into 5-FU, was tested as a novel strategy in gene-directed enzyme prodrug therapy against pancreatic cancer. In Panc1 and AsPc1 chemoresistant pancreatic cancer cells, wt H-1PV diminished the constitutive NF-κB activity. Moreover, NF-κB DNA binding and transcriptional activity were significantly reduced upon rPVH1-yCD/5-FC treatment. Importantly, the antitumor activity of wt H-1PV and rPVH1-yCD/5-FC is likely to be associated with attenuation of both NF-κB and Akt/phosphatidylinositol 3-kinase (PI3K) activity. Therefore, it is assumed that the oncolytic activity of H-1PV is linked to NF-κB. These observations suggest that inhibitors of these signaling pathways may increase the effectiveness of pancreatic cancer therapy [77].

### 5.2. RNA Viruses

#### 5.2.1. Encephalomyocarditis Virus (EMCV)

Encephalomyocarditis virus (EMCV), belonging to *Picornaviridae* family, is a mammalian non-enveloped, positive sense-(+)ssRNA virus, which rarely infects humans [78,79]. In preclinical studies, EMCV oncolytic properties were implemented in the treatment of patients with CCRCC, which is well known for its high resistance to chemotherapy and radiation [79].

In CCRCC cells, EMCV virulence can be impaired by NF-κB suppression. In vitro studies on 786-O cell line treated with JSH-23, which inhibits LPS-induced NF-κB nuclear translocation, but not IκB degradation [80], demonstrated that JSH-23 significantly diminished LPS-induced NF-κB nuclear translocation, as well as susceptibility to EMCV virulence. Similarly, IKKγ-deficient 786-O cells showed resistance to EMCV virulence [79].

Importantly, both CCRCC and 786-O cells are characterized by von Hippel–Lindau (VHL), a tumor suppressor protein, inactivation. The loss of VHL results in hypoxia-inducible factor (HIF)-2α stabilization at tumor enhancers. Thus, CCRCC genes linked to tumorigenesis are upregulated [81]. *VHL-null* 786-O cells infected with EMCV did not show IFN-mediated antiviral response, but enhanced survival signaling under control of NF-κB and elevated viral replication. Since VHL is a negative regulator of HIF, observed events can be a result of HIF-mediated promotion of NF-κB activation of the survival pathway. Thus, infected cells can be sensitized to virally induced cytotoxicity [79].

Importantly, the cross-talk between NF-κB signaling and IKK-regulated type I IFN gene expression is an obvious fact that implements the use of IKK inhibitors in oncolytic virotherapy. IKK-inhibitors, BMS-345541 [82] and TPCA-1 [83], counteract cell proliferation, p65 nuclear translocation, and NF-κB-regulated *CXCL8* gene expression in glioma cells. Both inhibitors, when combined with OVs, such as EMCV, block IFN-regulated antiviral responses since the reversion of IFN-induced anticythopathic effect and antiviral effect by IKK inhibitors in glioma cells can be observed. Particularly, BMS-345541 inhibits the action of IFN against EMCV, which exert a CPE in U87 cells. Importantly, the correlation between this effect and suppression of NF-κB by BMS-345541 was observed [84].

#### 5.2.2. M1 Alphavirus

M1 alphavirus, an ss(+)RNA virus and a member of *Togaviridae* family, is an arthropod-borne pathogen, which promotes apoptosis of glioma cells. M1 displays high tumor tropism and oncolytic properties in vitro, in vivo, and ex vivo [85,86]. Particularly, in order to induce apoptosis, M1 targets zinc-finger antiviral protein (ZAP)-deficient tumor cells and acts via endoplasmic reticulum (ER) [86,87]. Cyclic adenosine monophosphate (cAMP) activation is required for tumor cell sensitization toward M1 [86].

When combining M1 with H89, a protein kinase A (PKA) [88] and NF-κB transcriptional inhibitor [89], the antiviral response is inhibited and M1-induced oncolysis is elevated. In addition, M1-induced NF-κB p65 phosphorylation is abolished in colon HCT-116 and Capan-1 cancer cell lines.

This effect may result from the activation of exchange protein directly activated by cAMP isoform 1 (Epac1), which may either stabilize IκB or upregulate cellular oncogene Fos (c-Fos). c-Fos, in turn, interacts with NF-κB p65 and impairs its transcriptional activity [90]. Therefore, M1-induced ISGs expression can be abolished. Together, NF-κB has been shown to be involved in preventing type I IFN response by H89 [91].

#### 5.2.3. Newcastle Disease Virus (NDV)

The Malaysian field outbreak isolate, NDV strain AF2240, is an avian pathogen and a member of ss(-)RNA *Paramyxoviridae* family. AF2240 is regarded as a good and efficient candidate in oncolytic virotherapy due to its high immunogenicity and selectivity toward tumors [92]. In tumor cells, NDV triggers innate and adaptive immune responses and exerts cytotoxic effect via caspase activation resulting in apoptosis [85]. What is important, ER stress upon NDV treatment induces antitumor immunity [64].

AF2240 triggers p38 mitogen-activated protein kinase phosphorylation upstream of IκBα processing and NF-κB nuclear translocation in 786-O cells. NF-κB, in turn, regulates IFN-β production in the early phase of NDV infection. In 786-O cells harboring *VHL* gene, the activity of NF-κB, IFN-β secretion, and killing effect was elevated upon NDV infection in comparison with 786-O cells. Interestingly, in NDV-infected 786-O cells treated with JSH-23, a canonical NF-κB signaling inhibitor [80], NF-κB activation was not completely abolished. Therefore, a cross-talk is assumed between canonical and non-canonical NF-κB signaling upon oncolytic respiratory syncytial virus (RSV) infection suggesting other regulatory mechanisms [93].

#### 5.2.4. RV

RV is a human pathogen and enterotropic dsRNA virus belonging to *Reoviridae* family. RV is regarded as a good candidate for oncolytic treatment as an easily manufactured therapeutic agent, which has a stable genome and displays minimal toxicity for humans [94]. The ability of RV to use certain signaling pathways in transformed cells targeting constitutive Ras signaling makes it a good candidate as a drug against prostate cancer, glioma, MM, and pancreatic adenocarcinoma [25]. Indeed, an RV-based oncolytic Reolysin (Oncolytics Biotech Inc., Calgary, AB, Canada) has been approved as an orphan drug by FDA in ovarian, fallopian tube cancers, glioma, gastric, and pancreatic cancer [44].

In HeLa cells, RV activates p65/p50 complexes [95]. Further studies have shown that at late times, post infection RV inhibits NF-κB activation. This type of dynamics of NF-κB regulation in RV-infected cells is required for TNF-α- and TRAIL-induced apoptosis [96].

In RV-infected cells, caspase 8-mediated apoptosis can be accelerated by glycogen synthase kinase (GSK)-3ß inhibitors [97]. GSK-3ß, which has an impact on NF-κB-regulated genes [98], can be inhibited by AR-A014418 [99], which also counteracts RV-induced NF-κB activation in HCT116 cells. Therefore, GSK-3ß inhibitors could serve as therapeutic tools in NF-κB-mediated inflammation and cancer [97].

Nutlin-3a is another promising anticancer agent, which triggers p53 signaling in cells with wt p53 [100,101]. In p53^+/+^ wt HCT116 and osteosarcoma U2OS cells treated with RV and nutlin-3a, increased NF-κB level and enhanced apoptosis were observed. NF-κB inhibition, in turn, resulted in reduced cell death in RV-infected and nutlin-3a treated wt HCT116 cells [102]. This observation demonstrated that cytotoxicity of RV combined with nutlin-3a relies upon NF-κB. Importantly, NF-κB activation depends on p53 [103]. NF-κB, in turn, controls p53 target genes [104]. Taken together, both p53 and NF-κB act synergistically to induce apoptosis. These interactions are important in therapeutic management of cancers expressing wt p53 [102].

RV activates NF-κB p65 nuclear translocation not only in p53^+/+^ HCT116 cells, but also in cells lacking p53. Since NF-κB inhibition has a profound effect on reduction of cell death following RV infection, the enhancement of NF-κB activation to increase cell death was evaluated by combining RV and actinomycin-D (ActD) or etoposide (Etp) treatment of p53^+/+^ cells. Increased oncolysis observed may also be a consequence of upregulation of p21-encoding cyclin-dependent kinase inhibitor 1 (*CDKN1A*) gene [105], whose regulation depends on NF-κB [106].

Although RV-induced oncolysis has been implemented in clinical trials, the mechanisms of this process are still not fully elucidated. In breast cancer treatment studies, NF-κB has been investigated as a factor, which either promotes or suppresses apoptosis of tumor cells [107]. RV infection of MCF7 and hypotriploid HTB133 cells upregulated p65 NF-κB expression, as well as its nuclear translocation and DNA binding. NF-κB inhibition, in turn, resulted in oncolytic protection and downregulation of p53-upregulated modulator of apoptosis (PUMA) expression. This study suggests that in breast cancer therapy, NF-κB acts proapoptotically, mediates RV-induced oncolysis, and may serve as a predictive indicator of the sensitivity to RV treatment [108].

#### 5.2.5. RSV

RSV, a member of *Paramyxoviridae* family, is an ss(-)RNA virus, which is regarded as an oncolytic agent. However, the data on the effect of RSV on apoptosis in individual cell types are, in fact, conflicting. Nevertheless, the oncolytic potential of RSV toward certain tumors, including skin neoplasm, has been demonstrated [109].

As shown in human prostate tumor xenografts, RSV leads to oncolysis of prostatic cancer cells in vitro and in vivo. In PC-3 cells, RSV-induced apoptosis via an intrinsic pathway that involves caspase-3. NF-κB downregulation, observed upon RSV infection, results in the upregulation of pro-apoptotic genes and downregulation of anti-apoptotic genes. Consequently, apoptosis and oncolysis can be observed [110].

Further studies demonstrated that RSV-induced modulation of innate antiviral response is linked to androgen dependence and/or androgen receptor expression on target cells. PC-3 cells, which are androgen-independent, are susceptible to RSV-induced apoptosis despite functional IFN-mediated antiviral signaling, but a defective NF-κB response. Importantly, defective NF-κB activation could not be observed in androgen-sensitive LNCaP and RWPE-1 prostate cancer cells [111].

#### 5.2.6. VSV

VSV is a non-segmented ss(-)RNA virus, belonging to *Rhabdoviridae* family, infecting insects and livestock animals. Preexisting immunity against VSV in humans, as well as cell-cycle-independent cytoplasmic replication cycle without risk of host cell transformation in various cell lines, renders VSV a promising tool for oncolytic virotherapy [112]. Importantly, high sensitivity of VSV to IFN response favors it as a candidate for the treatment of tumors with aberrant IFN signaling. Nevertheless, VSV may also target tumors with abnormal function of Ras, p53, and myelocytomatosis (Myc) proteins [85].

The importance of IKK/NF-κB signaling in type I IFN-mediated antiviral response has been demonstrated in glioma cell lines. Both TPCA-1, an IKKβ inhibitor, which prevents IκBα phosphorylation [83], and a selective IKKα/IKKβ inhibitor, BMS-345541 [82], have been proposed in combination with VSV for anticancer therapies. In glioma cells, TPCA-1 and BMS-345541 inhibited NF-κB activation and *CXCL8* gene expression, as well as IFN-activated gene expression. BMS-345541 has been demonstrated to counteract antiviral IFN effect against VSV in glioma cells since VSV is considered as a promising tool in oncolytic therapies of malignant brain tumors. Importantly, this effect correlated with decreased NF-κB activity [84].

The oncolytic potential of VSV can also be modulated by vorinostat [113], a histone deacetylase inhibitor [114], which modulates NF-κB-dependent genes [115]. Vorinostat increases p65 NF-κB nuclear accumulation, DNA binding, and acetylation, while inhibiting IFN signaling in PC-3 cells. Subsequent NF-κB activation also induces certain genes linked to autophagy. Together, vorinostat enhances VSV replication in PC-3 cells and oncolysis. The effect of vorinostat on NF-κB signaling, autophagy, and oncolysis in VSV-infected cells was also demonstrated in DU145 prostate cell line and HCT116 cells [113]. Other studies have shown the synergy of curcumin, an anti-inflammatory agent and NF-κB inhibitor [116] and VSV infection in enhancing oncolysis of PC-3 cells. When treated with curcumin and then infected with recombinant M protein mutant VSV, rM51R-M, the cells became sensitized to VSV-induced apoptosis and showed the reduction on phospho (p)-NF-κB expression. At the same time, the decrease in NF-κB-dependent anti-apoptotic Bcl-extra-large (xL) protein content was observed [117].

NF-κB signaling is involved in MM development [2]. NF-κB activity is also induced by VSV infection of myeloma cells and may promote apoptosis and virus spread. On the contrary, NF-κB plays a prosurvival role during VSV infection to ensure viral replication. Inhibition of NF-κB by BMS-345541 led to attenuation of VSV replication in U266 and 5TGM1 MM cell lines. Bortezomib, a proteasomal inhibitor [118], which inhibits IκBα degradation and p65 nuclear accumulation [119], counteracted virally induced p65 nuclear protein expression in U266 nuclear extracts. Therefore, antagonistically acting bortezomib may not be profitable for the virus spread. Despite no effect on the increase of VSV titer upon NF-κB in vitro, in vivo studies in mouse MM model have shown that bortezomib is not able to inhibit intratumoral replication of VSV. Moreover, bortezomib enhances the therapeutic effect of oncolytic VSV [120] armed with murine IFN-β and NIS VSV-IFNβ-NIS [121].

## 6. Conclusions

Oncolytic virotherapy is a new promising therapeutic approach, which needs improvement to enhance the antitumor effect. Therefore, combined therapies using immunomodulating drugs, including NF-κB regulators, are considered in the treatments. The importance of the complexity of NF-κB activation in tumor cells and the interplay between OVs and NF-κB signaling is shown in this review. Since NF-κB is a key player in tumorigenesis, which shapes the cellular response and plays a role in balancing the immune system, NF-κB activation level should be considered in oncolytic treatment. Thus, NF-κB activation level can indicate the efficacy and effectiveness of novel therapeutic approaches and help obtain the desired therapeutic effect.

## Figures and Tables

**Table 1:** Oncolytic DNA and RNA viruses in clinical trials. A selected list includes viruses described in the text.

OV	Treatment	Condition	Status	Phase	References
AV	Ad5-yCD/mutTKSR39rep-ADP (Theragene^®^)	Non-small cell lung cancer stage I	Recruiting	I	NCT03029871
	5-FC				
	vGCV				
	SBRT				
	Ad5-yCD/mutTKSR39rep-hIL12	Prostate cancer	Recruiting	I	NCT02555397
	Ad5-yCD/mutTKSR39rep-hIL12	Metastatic pancreatic cancer	Recruiting	I	NCT03281382
	5-FC				
	ADV/HSV-tk	Metastatic non-small cell lung cancer Metastatic triple-negative breast cancer	Recruiting	II	NCT03004183
	Valacyclovir				
	SBRT				
	CG0070	Bladder cancer	Active, not recruiting	II	NCT02365818
	DNX-2401 (Delta-24-RGD-4C)	Brainstem glioma	Recruiting	I	NCT03178032
	Neoadjuvant therapy				
	DNX-2401 (Delta-24-RGD-4C)	Glioblastoma	Recruiting	II	NCT02798406
	Pembrolizumab	Gliosarcoma			
	DNX-2440	Recurrent glioblastoma	Recruiting	I	NCT03714334
	LOAd703	Biliary carcinoma	Recruiting	I/II	NCT03225989
		Colorectal cancer			
		Ovarian cancer			
		Pancreatic adenocarcinoma			
	LOAd703	Pancreatic cancer	Recruiting	I/II	NCT02705196
	Gemcitabine				
	Nab-paclitaxel				
	NSC-CRAd-S-pk7	Malignant glioma	Recruiting	I	NCT03072134
	OBP-301 (Telomelysin)	Melanoma stage III-IV	Recruiting	II	NCT03190824
	ONCOS-102	Castration-resistant prostate cancer	Recruiting	I/II	NCT03514836
	Cyclophosphamide				
	DCVac/PCa				
	ONCOS-102	Advanced or unresectable melanoma progressing after PD-1 blockade	Recruiting	I	NCT03003676
	Cyclophosphamide				
	Pembrolizumab				
	ONCOS-102	Malignant pleural mesothelioma	Recruiting	I/II	NCT02879669
	Cyclophosphamide				
	Pemetrexed/cisplatin (carboplatin)				
	VCN-01	Locally advanced solid tumors	Recruiting	I	NCT02045602
	Gemcitabine	Metastatic solid tumors			
	Abraxane^®^	Pancreatic adenocarcinoma			
HSV-1	C134	Recurrent malignant glioma	Not yet recruiting	I	NCT03657576
	G207	Supratentorial neoplasms, malignant	Recruiting	I	NCT02457845
	M032 (NSC 733972)	Recurrent malignant glioma	Recruiting	I	NCT02062827
	rQNestin34.5v.2 (rQNestin)	Recurrent malignant glioma	Recruiting	I	NCT03152318
	Cyclophosphamide				
	TBI-1401(HF10) (Canerpaturev)	Pancreatic cancer stage III-IV	Recruiting	I	NCT03252808
	Gemcitabine				
	Nab-paclitaxel				
	TS-1				
	TBI-1401(HF10) (Canerpaturev)	Melanoma stage III-IV	Active, not recruiting	II	NCT03153085
	Ipilimumab				
	T-VEC	Basal cell carcinoma	Recruiting	I	NCT03458117
		Cutaneous lymphoma			
		Merkel cell carcinoma			
		Non-melanoma skin cancer			
		Squamous cell carcinoma			
		Recurrent breast cancer that cannot be removed by surgery	Active, not recruiting	II	NCT02658812
	T-VEC	Metastatic, unresectable, or recurrent HER2- negative breast cancer	Not yet recruiting	I	NCT03554044
	Anastrozole				
	Exemestane				
	Fulvestrant				
	Letrozole				
	Paclitaxel				
	Tamoxifen				
	T-VEC	Locally advanced or metastatic rectal cancer	Recruiting	I	NCT03300544
	Capecitabine				
	Fluorouracil				
	Oxaliplatin				
	T-VEC	Refractory T cell and NK cell lymphomas Cutaneous squamous cell carcinoma Merkel cell carcinoma Other rare skin tumors	Recruiting	II	NCT02978625
	Nivolumab				
	T-VEC Paclitaxel	Breast cancer Ductal carcinoma Invasive breast carcinoma Invasive ductal breast carcinoma	Recruiting	I/II	NCT02779855
	T-VEC	Melanoma stage III-IV	Recruiting	II	NCT02965716
	Pembrolizumab				
PV	H-1PV (ParvOryx™)	Metastatic inoperable pancreatic cancer	Recruiting	I/II	NCT02653313
RV	REOLYSIN^®^	KRAS mutant metastatic colorectal cancer	Active, not recruiting	I	NCT01274624
	Irinotecan				
	Leucovorin				
	5-FU				
	Bevacizumab				
	Wild-type Reovirus	Relapsed or refractory multiple myeloma	Active, not recruiting	I	NCT02514382
	Bortezomib				
	Dexamethasone				
	Wild-type Reovirus	Recurrent plasma cell myeloma	Not yet recruiting	I	NCT03605719
	Carfilzomib				
	Dexamethasone				
	Nivolumab				
	Pomalidomide				
	Wild-type Reovirus Paclitaxel	Recurrent fallopian tube carcinoma Recurrent ovarian carcinoma Recurrent primary peritoneal carcinoma	Active, not recruiting	II	NCT01199263
VSV	VSV-hIFNβ-NIS	Stage IV or recurrent endometrial cancer	Recruiting	I	NCT03120624
	Technetium Tc-99m Sodium Pertechnetate				
	VSV-IFNβ-NIS	Malignant solid tumor	Recruiting	I/II	NCT02923466
	VSV-IFNβ-NIS and Avelumab				
	VSV-IFNβ-NIS	Refractory non-small cell lung cancer or Hepatocellular carcinoma	Not yet recruiting	I	NCT03647163
	Pembrolizumab				

Abbreviations: 5-FC, 5-fluorocytosine; 5-FU, 5-fluorouracil; AV, adenovirus; HER2, human epidermal growth factor receptor 2; hIFNβ, human interferon β; hIL12, human interleukin-12; HSV-1, herpes simplex virus type 1; NIS, sodium iodide symporter; NK, natural killer; OV, oncolytic virus; PD-1 programmed cell death protein 1; PV, parvovirus; RGD, arginine-glycine-aspartic acid; RV, reovirus; SBRT, stereotactic body radiation therapy; tk, thymidine kinase; T-VEC, Talimogene laherparepvec; vGCV, valganciclovir; VSV, vesicular stomatitis virus; yCD, yeast cytosine deaminase.
